# A Positive Outcome and Literature Review of Acute Myeloid Leukaemia in Pregnancy

**DOI:** 10.7759/cureus.93974

**Published:** 2025-10-06

**Authors:** Saskia Craine, Harry King

**Affiliations:** 1 Obstetrics and Gynaecology, Blackpool Teaching Hospitals, Blackpool, GBR; 2 Cardiology, Blackpool Teaching Hospitals, Blackpool, GBR

**Keywords:** acute myeloid leukaemia, cancer, chemotherapy, pregnancy, remission, treatment

## Abstract

Acute myeloid leukaemia (AML) in pregnancy is rare and difficult to manage. Here, we describe a case of AML with *NPM1* mutation. The patient was counselled regarding the therapy-attributable risks, underwent an elective caesarean section at 33 weeks’ gestation, and delivered a morphologically normal fetus. As per WHO guidance, AML treatment should not be delayed. Management requires close collaboration with obstetric and neonatal teams, striking a balance between maternal and foetal survival. Counselling on elective termination should be provided. Treatment decisions should reflect gestational age at diagnosis, maternal tolerance, and drug toxicity. The aim is to complete pregnancy with a viable, healthy fetus and with minimal harm to the mother.

## Introduction

Acute myeloid leukaemia (AML) is a rare but aggressive haematologic malignancy with an incidence of 1 in 75,000 pregnancies [1,2]. AML can result in maternal leucocytosis, thrombosis, and coagulopathy, each of which contributes to increased maternal mortality [3]. In pregnancy, leukaemia heightens foetal risks, including abortion, intrauterine growth restriction, and perinatal mortality. These foetal risks are partly due to increased susceptibility to infections from maternal pancytopenia and reduced placental perfusion [4]. Management is guided by gestational age: early pregnancy may require counselling for termination owing to treatment-related teratogenicity, whereas chemotherapy in later trimesters is considered safer for the foetus but still carries risks such as stillbirth and low birthweight [1].

Building on this background, we describe a case of AML with *NPM1* mutation diagnosed during pregnancy and managed in our department. The patient received multidisciplinary care, underwent an elective caesarean delivery at 33 weeks, and delivered a morphologically normal infant. This case illustrates that, with timely treatment and coordinated obstetric, haematologic, and neonatal management, favourable maternal and neonatal outcomes are achievable.

## Case presentation

A 41-year-old woman, gravida 6 para 2, at 18 weeks’ gestation, was diagnosed with AML after presenting unwell post-dental extraction. Routine bloods revealed a white blood cell count of 19.2 × 10⁹/L (normal range: 4.0-11.0 × 10⁹/L), raising suspicion for AML.

Cytogenetic analysis was unsuccessful, and fluorescence in situ hybridisation detected no chromosomal abnormalities. Next-generation sequencing identified *NPM1* and *IDH2* mutations, while the *FLT3* mutation was absent. Significant HLA antibodies were present. A peripheral blood film confirmed AML with an *NPM1* mutation.

She was advised to continue folic acid and vitamin D supplementation for pregnancy and was managed by a multidisciplinary team involving haematology and obstetrics. After detailed counselling regarding the risks of chemotherapy during pregnancy, potential maternal and foetal complications, and treatment alternatives, she provided informed consent for induction chemotherapy with daunorubicin and cytarabine (DA 3+8). She tolerated induction therapy without complication and achieved morphological remission. She received supportive therapies, including amphotericin B, granulocyte colony-stimulating factor, platelet transfusions, and central venous catheter placement.

At 24 weeks, an anomaly scan was normal. Serial foetal monitoring occurred due to potential intrauterine growth restriction. At 33 weeks, an elective caesarean section was performed for AML (blood loss of 250 mL). The preterm infant needed oxygen for 48 hours, then self-ventilated. By day six, the neonate was fully established on feeds with preterm formula and iron supplementation.

With a venous thromboembolism risk score of 8, she received antenatal and postoperative thromboprophylaxis. Postpartum haemoglobin was 85 g/L (reference range: 121-151 g/L), and iron supplementation was not advised.

Two weeks postoperatively, she developed a wound infection requiring suture removal in theatre, intravenous antibiotics, and prophylactic oral antibiotics. Microbiological cultures grew *Enterococcus faecalis* (from a wound swab) and *Staphylococcus hominis* (from a blood culture). Consequently, her consolidation chemotherapy (FLAG-Ida) was delayed by four weeks.

She also experienced ongoing per vaginal bleeding, managed with tranexamic acid, norethisterone, and multiple platelet transfusions to maintain counts above 30 × 10⁹/L (reference range: 150-450 × 10⁹/L). Two months following FLAG-Ida, minimal residual disease was detected. She subsequently underwent an allogeneic bone marrow transplant, after which her care was continued at Manchester Royal Infirmary. Post-transplant, she experienced multiple episodes of neutropenic fever and developed graft-versus-host disease. Her child continues to grow and develop without adverse outcomes.

## Discussion

The occurrence of AML in pregnancy is rare, and early features, such as fatigue, dyspnoea, anaemia, and thrombocytopenia, may be misattributed to normal pregnancy, leading to a delayed diagnosis. Untreated, AML can lead to rapid maternal and foetal mortality, and delaying induction of chemotherapy reduces the likelihood of remission [5]. The standard induction therapy combines cytarabine and anthracycline for induction to achieve complete remission. Treatment should be discussed with the patient and individualised according to gestational age. The remission rates for pregnant women are currently 70-75%, with median survival time influenced by several factors, including cytogenetic abnormalities [6]. Significantly longer survival was observed when induction therapy was not delayed and treatment was received before delivery [6]. The decision to commence or delay chemotherapy must balance maternal and foetal survival with the risk of treatment-related morbidity. The management recommendations are outlined in Table 1 [6]. Treatment decisions are shaped by multiple factors, including leukaemia subtype, disease severity, and the patient’s values and preferences.

**Table 1:** Recommendations for the management of acute myeloid leukaemia (AML) and acute promyelocytic leukaemia (APL) in pregnancy.

Type of leukaemia	First trimester	Second Trimester	Third trimester
AML	Pregnancy termination, then conventional therapy	Cytarabine + doxorubicin	Cytarabine + doxorubicin
APL	Pregnancy termination, then conventional therapy	All trans-retinoic acid + anthracycline	All trans-retinoic acid + anthracycline

**Figure 1:**
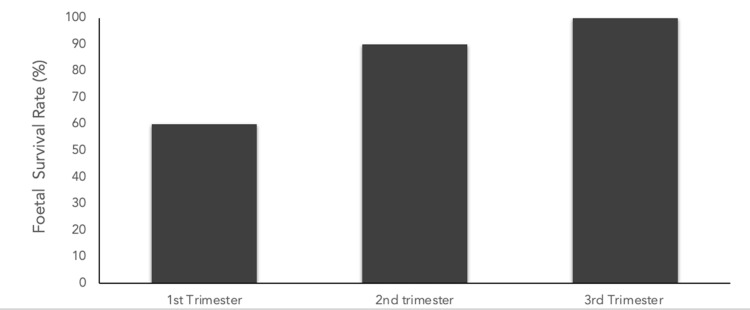
Bar chart demonstrating the average foetal survival rate (%) for each trimester using data from Chelghoum et al.

A diagnosis of AML in the first trimester is associated with poor pregnancy outcomes, including a high risk of spontaneous loss and foetal malformations due to the requirement for intensive chemotherapy. Chelghoum et al. reported foetal death in ~40% of cases treated with chemotherapy in the first trimester, compared with ~10% in the second. Almost all infants exposed in the third trimester were live births without significant anomalies (Figure 1) [7]. The literature emphasises the importance of effective treatment, which significantly improves survival rates.

The management of AML should be multidisciplinary, incorporating obstetrics, neonatal care, and haematology. The delivery is ideally planned between 35 and 37 weeks’ gestation, which remains the primary goal [3]. Survival rates exceed 90% beyond 28 weeks’ gestation and above 95% beyond 32 weeks in large centres [1]. Chemotherapy should not be administered after the 35th week of gestation [3]. Throughout treatment, intensive ultrasound monitoring is required to monitor foetal growth, cardiac function, and placental status. Induction chemotherapy during the second trimester (13-24 weeks) should be considered, allowing continuation of pregnancy after careful counselling. Ultimately, adaptations to therapy must reflect gestational age, maternal tolerance, and drug toxicity with a primary objective of completing pregnancy, with a viable neonate while minimising maternal risk.

## Conclusions

AML during pregnancy presents significant therapeutic challenges requiring multidisciplinary management. In this case, timely treatment and coordinated obstetric and neonatal care facilitated elective delivery at 33 weeks, resulting in favourable maternal and neonatal outcomes. This highlights the importance of early diagnosis, prompt therapy, and individualised treatment planning in achieving successful results.

## References

[1] Patel SJ, Ajebo G, Kota V, Guddati AK (2020). Analysis of outcomes in hospitalized pregnant patients with acute myeloid leukemia. Am J Blood Res.

[2] Pavlidis NA (2002). Coexistence of pregnancy and malignancy. Oncologist.

[3] Fracchiolla NS, Sciumè M, Dambrosi F (2017). Acute myeloid leukemia and pregnancy: clinical experience from a single center and a review of the literature. BMC Cancer.

[4] Avivi I, Brenner B (2014). Management of acute myeloid leukemia during pregnancy. Future Oncol.

[5] Zhu D, Tang D, Chai X, Zhang G, Wang Y (2021). Acute leukemia in pregnancy: a single institutional experience with 21 cases at 10 years and a review of the literature. Ann Med.

[6] Thomas X (2015). Acute myeloid leukemia in the pregnant patient. Eur J Haematol.

[7] Chelghoum Y, Vey N, Raffoux E (2005). Acute leukemia during pregnancy: a report on 37 patients and a review of the literature. Cancer.

